# Progress in Our Understanding of the Cross-Protection Mechanism of CTV-VT No-SY Isolates Against Homologous SY Isolates

**DOI:** 10.3390/pathogens14070701

**Published:** 2025-07-16

**Authors:** Grazia Licciardello, Giuseppe Scuderi, Marcella Russo, Marina Bazzano, Giuseppe Paradiso, Moshe Bar-Joseph, Antonino F. Catara

**Affiliations:** 1 CREA—Council for Agricultural Research and Economics, Research Centre for Olive, Fruit and Citrus Crops, 95027 Acireale, CT, Italy; giuseppe.paradiso@crea.gov.it; 2 Agrobiotech Soc. Coop., z.i. Blocco Palma I, Stradale Lancia 57, 95121 Catania, CT, Italy; gscuderi@agrobiotech.it (G.S.); mrusso@agrobiotech.it (M.R.); mbazzano@agrobiotech.it (M.B.); 3 The S. Tolkowsky Laboratory, Department of Plant Pathology, The Volcani Center, Agriculture Research Organization, Bet Dagan, Rishon Lezion 7528809, Israel; mbjoseph@gmail.com; 4 Formerly of Department of Phytosanitary Science and Technologies, University of Catania, 95123 Catania, CT, Italy; acatara@agrobiotech.it

**Keywords:** superinfection exclusion, real-time RT-PCR, polymorphism, seedling yellows, decline

## Abstract

The *Citrus tristeza virus* (CTV), a member of the *Closterovirus* genus, is considered a serious threat to citrus trees grafted onto sour orange (SO) rootstock. In the Mediterranean area, the most prevalent CTV strains are VT and T30. The VT strain includes both mild and severe isolates, some of them associated with seedling yellows (SY) syndrome. Mild CTV-VT isolates that do not induce SY symptoms (no-SY) show minor variations in their Orf1a, p23, and p33 genes, with a single nucleotide polymorphism at position 161 of the p23 gene. These isolates can repress superinfection with homologous severe isolates. The aim of this study was to investigate the mechanism of cross-protection by means of biological indexing, real-time RT-PCR high-resolution melting (HRM), and p23 gene amplicon sequencing. Four no-SY CTV-VT isolates were inoculated onto SO seedlings and Hamlin sweet orange trees grafted on SO. These plants were later challenged with two homologous CTV-VT SY isolates and remained asymptomatic. The biological evaluation of the infection process in superinfected plants was investigated via inoculation of the bark on SO seedlings that were also asymptomatic. A parallel HRM analysis of midvein RNA extracts revealed that the melting temperature (Tm) of the no-SY isolates was statistically lower than that of the SY isolates. The Tm values of RNAs extracts from superinfected plants were not statistically different from those of the no-SY isolates. This suggests that the SY isolates failed to establish infection or replicate in plants pre-inoculated with no-SY isolates. This blockage of replication resembles superinfection exclusion, with attractive perspectives to prevent SY damage in field applications.

## 1. Introduction

*Citrus tristeza virus* (CTV) is a phloem-limited, flexible, filamentous *Closterovirus* (2000 × 11 nm) [1,2] that severely affects citrus trees grafted onto sour orange (*Citrus aurantium*, SO) rootstock. Alternative rootstocks can reduce the damage, though some have horticultural limitations and shorter lifespans in certain soils [3]. Sour orange is still considered one of the most important citrus rootstocks in many Mediterranean countries.

The 19.3 kb single-stranded positive-sense RNA genome of CTV contains twelve open reading frames (ORFs) and the 5′ and 3′ termini [4]. The virus is spread through infected budwood propagation and naturally by certain aphid species, primarily *Aphis* (*Toxoptera*) *citricidus* and *A. gossypii* [5,6]. CTV isolates are categorized into at least eleven genotypes—T3, T30, T36, T68, VT, RB, HA16-5, S1, L1, M1, and A18—based on their full genome sequences [7]. Of the 127 fully sequenced isolates, around 35 isolates are from the Mediterranean area, including Italy, Montenegro, and Iran (data retrieved from www.ncbi.nlm.nih.gov, 8 March 2025).

CTV infection can induce three major syndromes: tristeza decline (QD), stem pitting (SP), and seedling yellows (SY). The symptoms vary with the host, rootstock–scion combination, tree age, and environmental conditions. Tristeza decline severely affects sweet orange (*C. sinensis*), grapefruit (*C. paradisi*), and mandarin (*C. reticulata*) grafted onto SO. Stem pitting, which causes xylem pits in Mexican lime (*C. aurantifolia*), alemow (*C. macrophylla*), grapefruit, and sweet orange, is associated with stunted growth and reduced fruit yield [3]. SP isolates affecting sweet orange have been detected only sparsely in Mediterranean regions. 

SY syndrome, caused by CTV strains T36 [8] and some VT isolates [9], primarily affects lemon, grapefruit, and SO seedlings grown in greenhouses, inducing severe chlorosis and stunting. However, infected seedlings may recover after being transplanted into the field [3,5]. The ectopic expression of the p23 gene in CTV has been associated with its ability to induce CTV-like symptoms in various Citrus species but not in *Nicotiana* spp. [8,10,11]. The p23 protein is unique to CTV, featuring a zinc finger domain and nucleolar signal, likely evolved for interaction with citrus hosts [12]. It also mediates asymmetric RNA strand accumulation and suppresses RNA silencing within cells [13,14]. 

Cross-protection against CTV SP is successfully employed in Brazil, South Africa, and Australia [15,16], provided that the mild protective strains are genetically similar to the local strains. Using an infectious cDNA clone of a T36 isolate, Folimonova et al. [17] demonstrated that isolates of the same CTV genotype exhibit superinfection exclusion (SIE). However, attempts to select protective strains based on visual symptoms have proven unsuccessful [18,19,20].

Recent studies on SP induction by the T68 genotype [21] have shown that minor nucleotide variations in specific regions significantly influence disease outcomes. Similarly, recovery from SY is linked to stable mutations in just a few nucleotides, generating no-SY variants [9]. Sweet orange grafted onto SO and inoculated with no-SY VT variants does not develop SY symptoms and suppresses homologous SY isolates upon superinfection [22]. A sequence analysis revealed that no-SY VT isolates that were nearly identical to an SY-inducing VT isolate, differing only by eight amino acid substitutions within p33, p23, and Orf1a, resulted in symptomless infections in SO [9,22]. A single-nucleotide mutation in the p23 gene (nucleotide position 161) resulted in an amino acid change from serine to asparagine (position 54) and was possibly responsible for the shift from an SY to a no-SY phenotype [22]. However, isolates from different strains (e.g., mild T30 vs. SY VT) do not interact biologically [22]. Additional research showed that simultaneously inoculated mild and severe CTV strains with different genotypes (T30 and SY568) did not interfere with each other′s establishment in sweet orange, with the severe strain dominating both transcriptionally and biologically [23].

Multiple efforts to genetically distinguish mild and severe CTV variants have included bi-directional RT-PCR of a polymorphic p23 gene region [24] and conformational polymorphism analysis of the specific p18, p23, and p27 genes via single-strand conformation polymorphism analysis (SSCP) [25], capillary electrophoresis SSCP (CE-SSCP) [26], or a probe microarray in a lab-on-chip system [27]. The results were inconclusive. More recently, high-throughput sequencing (HTS) of the entire genomes has allowed for differentiation of the genotypes and variants [28,29,30,31]. Despite HTS being promising for the detection of potential cross-protective isolates [32], its use in routine diagnostics is still limited by its cost.

High-resolution melting (HRM) analysis is a method widely used for citrus plant traceability [33,34] and human pathogen mutant detection [35]. This method, based on the analysis of real-time RT-PCR amplicons, stained with fluorescent dye, exploits sequence-dependent differences in DNA melting behavior [36]. It has been successfully used in plant pathology to differentiate grapevine viruses [37], citrus viroid variants [38], and various bacterial and fungal genotypes [39,40,41]. 

This study aims to enhance our understanding of the mechanism of cross-protection by means of biological indexing, real-time RT-PCR high-resolution melting (HRM), and p23 gene amplicon sequencing. Indexing of superinfected plants with bark patches grafted onto SO seedlings, along with parallel real-time RT-PCR HRM analysis, clearly demonstrates that the cross-protection is based on the exclusion of SY isolates in plants pre-inoculated with selected no-SY isolates.

## 2. Materials and Methods

### 2.1. Selection of SY and No-SY Isolates

CTV isolates of the VT genotype were collected from citrus orchards and nurseries in eastern Sicily, some of which were previously used in cross-protection trials [9,22]. The selection process involved ELISAs, real-time PCR for genotyping, biological indexing on various citrus hosts, and genome sequencing via HTS [22]. The CTV-VT SY isolates induced severe symptoms on SO under greenhouse conditions, as well as stem pitting on Mexican lime and *C. macrophylla*, while the no-SY isolates did not cause symptoms on SO (Table 1). None of the isolates induced SP on SO or sweet orange (SwO) grafted onto SO. The no-SY isolates M39D, M101, and M55 were originally observed in macrophylla seedlings in a naturally infected nursery. Isolate Q7 was sourced from a 30-year-old Tarocco Lempso sweet orange tree, while Nan1 came from a 30-year-old Moro SwO tree. The SY isolates were all collected from commercial plots: P1R1 from a 30-year-old Tarocco S. Alfio tree, Nan3 from a 40-year-old Moro tree, and P7 from a young Pineapple tree grafted onto *C. macrophylla* inoculated with the SG29 isolate [22]. According to the HTS sequencing analysis, the SY and no-SY isolates showed a high level of nucleotide identity (99%) [22].

The no-SY and SY isolates collected during the surveys were tested under greenhouse conditions (18–30 °C) on various citrus and other rutaceous, including conventional indicators to detect the co-presence of viruses and viroids. 

### 2.2. Cross-Protection Trials on Sour Orange and Sweet Orange on SO Plants

The cross-protection trials began in June 2020 on SO seedlings and Hamlin SwO plants grafted onto SO (H/SO) with pre-inoculation of the no-SY M39D isolate, followed by superinfection with the SY P7 isolate (Table S1). Other trials included bark graft inoculation of the Q7, Nan1, and M101 no-SY isolates onto SO seedlings and H/SO sweet oranges on SO, followed by superinfection with the SY isolates P7 and P1R1 (Table S1).

The plants were inoculated in the greenhouse with bark patches of the no-SY isolate; after the inoculated plants became ELISA-positive, they were challenged with the SY isolate. The P7 SY isolate was inoculated onto H/SO two months post-infection with no-SY M39D, whereas SO seedling inoculation was performed six months post-infection with no-SY Q7. All the plants were transferred to unprotected conditions in 50 L containers. An additional test was performed in the greenhouse by inoculating 18-month-old SO with Nan1 no-SY and superinfecting it with P7. M101 no-SY was challenged with P1R1 SY.

Two years after the challenge inoculation, in March 2023, the replication of CTV in all the plants superinfected with the SY isolates was investigated by means of biological indexing on SO. The tests were conducted via the inoculation of two SO seedlings from each source plant with two pieces of bark, visual monitoring, ELISA, real-time RT-PCR, and HRM analysis of the p23 gene. The evaluation was extended to some plants that had been inoculated years before and maintained in open air [22]. 

### 2.3. Primer Design for RT-PCR HRM Assay 

The HRM assay to discriminate VT SY and no-SY CTV isolates targeted a single-nucleotide polymorphism located at nucleotide position 161 of the p23 gene [22]. Specific primer pairs were designed on the nucleotide sequences of the p23 gene from VT SY and no-SY isolates from our collection. The sequences underwent multiple alignment using MEGA X [42], and primers P23-CP-HRM-F5′-GAATCCCTTCATTATCGACGC-3′ and P23RevHRM3 5′-ACTCCTATTATTCTCGCGCG-3′ were designed using PrimerPremier 6 (PREMIER Biosoft, San Francisco, CA, USA), encompassing the 161 nt polymorphism and producing an amplicon 78 bp in size.

The variability of the G/A polymorphism at nucleotide position 161, which induces a Ser/Asp change at the aminoacidic level, was investigated in three VT SY and six no-SY isolates from our collection (SG29, Nan3, Mac1a, M39, M39D, Mac55, Q7, Mac101, Nan1), as well as in seven isolates representative of the main genotypes retrieved from GenBank (VT: EU937519; T318A, DQ151548; T30: AF260651; T385, Y18420; T3: KC525952; T36: EU937521; RB: MZ8703549) (Table S2), and aligned using MEGA X [42].

### 2.4. RNA Isolation and cDNA Synthesis 

Total RNA was extracted from 100 mg of bark tissue or roots using Trizol^®^ reagent (Thermo Fisher Scientific, Waltham, MA, USA) in accordance with the manufacturer′s protocol and subsequently quantified using a Nanodrop^®^ ND-100 (Thermo Fisher Scientific, Waltham, MA, USA). cDNA synthesis was performed using the High-Capacity cDNA Reverse Transcription Kit (Thermo Fisher Scientific, Waltham, MA, USA). The reaction mixture contained 1X RT-Buffer, 2 mM dNTPs, 2 mM random primers, MultiScribe Reverse Transcriptase (Thermo Fisher Scientific, Waltham, MA, USA), RNaseOUT recombinant ribonuclease inhibitor (Thermo Fisher Scientific, Waltham, MA, USA), 500 ng RNAs, and DEPC-treated H_2_O to achieve a final volume of 20 µL. The samples were incubated at 25 °C for 10 min, 37 °C for 2 h, and 25 °C for 5 s in a Veriti^TM^ 96-Well fast thermal cycler (Thermo Fisher Scientific, Waltham, MA, USA).

### 2.5. Real-Time RT-PCR and HRM Assay

A 4 μL aliquot of cDNA (25 ng/μL) underwent PCR in a 25 μL reaction mixture containing Buffer 1x MgCl_2_ 1.5 mM, dNTPs 0.2 mM, SYTO 9 1.67 μM, P23-CP-HRM-F 0.1 μM, P23RevHRM3 0.1 μM, Biotaq DNA Pol. 1 U/μL (Meridian Bioscience, Milan, Italy), and water to a total volume of 11 μL. The samples were analyzed in triplicate.

The reaction was steered in a StepOnePlus (Applied Biosystem, Waltham, MA, USA) instrument with the following cyclic conditions: a denaturation step at 95 °C for 10 min, followed by 40 cycles at 95 °C for 15 s and annealing at 62 °C for 1 min. Final extension was performed at 72 °C for 7 min. The high-resolution melting curves of the PCR amplicons were obtained with temperatures ranging from 95 °C for 15 s to 62 °C for 1 min and 95 °C for 15 s with a 0.1 °C increase in temperature every two seconds. The melting curves were analyzed using High-Resolution Melt Software v3.2 (Applied Biosystems TM, Waltham, MA, USA). One-way ANOVA followed by Student–Newman–Keuls post-hoc test was analyzed on the mean values of melting temperatures (Tm). Statgraphics PLUS v5.1 software (StatPoint Technologies, Inc., Warrenton, VA, USA) at confidence level of 99% (α = 0.01) was used.

### 2.6. High-Throughput Amplicon Sequencing of the p23 Gene

The P23HRMfw1-TTTCTGTGAACCTTTCTGACG and P23R-TTGTTGACYTTACAYTTACG TTC primer set was used to amplify a 238 bp fragment of the p23 gene from the total RNAs extracted from six representative superinfected plants from trials ‘Seq’ and ‘H/AA’. The amplicons were generated in a 25 µL reaction mixture containing Buffer PCR Rxn (Thermo Fisher Scientific, Waltham, MA, USA ) 1x, MgCl_2_ 3 mM, dNTPs 0.4 mM, primers 0.2 µM, Platinum taq DNA polymerase 1 Unit (Thermo Fisher Scientific, Waltham, MA, USA), Superscript II reverse transcriptase (Thermo Fisher Scientific, Waltham, MA, USA) 1 µL, RNaseOUT recombinant ribonuclease inhibitor (Thermo Fisher Scientific, Waltham, MA, USA) 1 µL, and RNA (50 ng/µL) 4 µL. The reaction was conducted in a VeritiTM 96-Well fast thermal cycler (Applied Biosystem, Waltham, MA, USA) with the following cyclic conditions: a denaturation step at 98 °C for 5 min, followed by 20 cycles at 98 °C for 30 s and annealing at 58 °C for 30 s, and final extension at 72 °C for 5 min. 

Post-amplification quality control was performed on a Bioanalyzer (Agilent Technologies, Santa Clara, CA, USA) by Cogentech s.r.l. (Milan, Italy). Target-enriched Illumina paired-end multiplexed sequencing libraries were prepared using the SureSelect XT HS2 DNA System (Agilent Technologies, Santa Clara, CA, USA), following the manufacturer′s instructions. Libraries were purified with AMPure XP beads (Beckman Coulter Life Sciences, Brea, CA, USA), and their quality was assessed using the Agilent TapeStation system (Agilent Technologies, Santa Clara, CA, USA).

Sequencing was performed on a NovaSeq 6000 Dx instrument (Illumina, San Diego, CA, USA). Sequence reads were aligned to the p23 reference gene using the BWA-MEM aligner [43]. Aligned reads were then filtered to retain only mapped reads using samtools view -F 4 [44], and subsequently sorted with samtools sort. Variant calling at nucleotide position 161 was performed using bcftools mpileup [45], generating a Variant Call Format (VCF) file reporting nucleotide frequencies and coverage. For alignment visualization, a random subset of 100K mapped reads was sampled using samtools view and indexed with samtools index [44]. 

## 3. Results

### 3.1. Symptomatic Reactions of Sour Orange Seedlings and Sweet Orange Grafted onto SO, Inoculated with No-SY and SY Isolates

**Figure 1:**
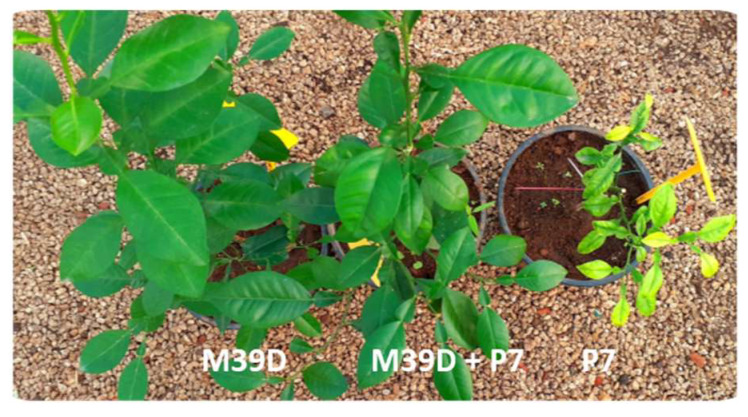
Sour orange seedlings inoculated with the M39D no-SY CTV-VT isolate showed no symptoms, even after superinfection with the P7 SY isolate (M39D + P7). On the contrary, strong SY symptoms are clearly visible on SO seedlings inoculated with the P7 isolate. This picture was taken about 6 months after the challenge inoculation.

**Figure 2:**
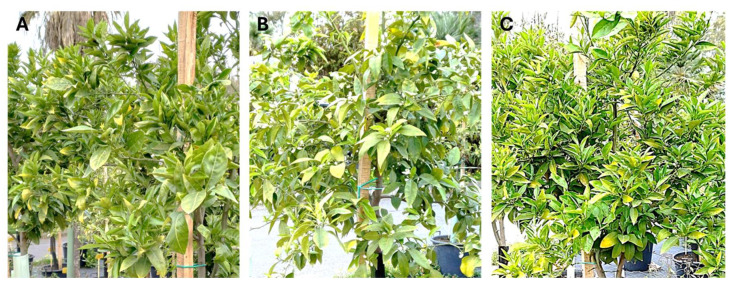
Hamlin sweet orange trees grafted onto sour orange showing no symptoms after four years: (**A**) a tree inoculated with the M39D no-SY isolate; (**B**) a tree inoculated with M39D and challenged with the P7 SY isolate two months later; (**C**) a control tree (non-inoculated).

Sour orange seedlings pre-inoculated with no-SY isolates and superinfected with an SY isolate grew normally, while those inoculated with P7 SY without pre-inoculation yellowed and declined in a short time (Figure 1). The height and canopy of H/SO plants and SO seedlings inoculated with only the no-SY isolate and those of non-inoculated plants grown in the open air in containers were very similar four years after inoculation. Sweet orange plants grafted onto SO grew less vigorously than the sour orange seedlings, without significant differences among the superinfected, mild-inoculated, and control plants (Figure 2). Some plants (inoculated or not) showed sparse and mild foliar zinc and manganese deficiencies due to the soil composition and container growth limits. 

None of the SO seedlings inoculated with bark from the plants initially infected with no-SY isolates and then superinfected with SY isolates showed any of the symptoms associated with P7 and P1R1 SY infection, such as leaf yellowing, size reduction, short internodes, or decline. Nonetheless, ELISA results showed a positive reaction, suggesting no-SY infection and a lack of SY infection.

### 3.2. Analysis of G/A Nucleotide Polymorphism of p23 Gene Among Different CTV Strains

**Figure 3:**
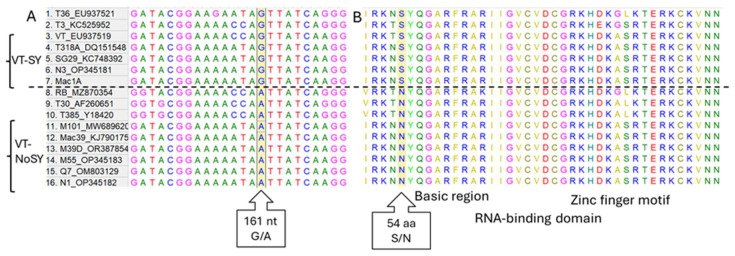
Multiple alignment at the nucleotide (**A**) and aminoacidic (**B**) levels showing partial p23 gene encompassing the G/A polymorphism located at nucleotide position 161, corresponding to an S/N aminoacidic change at the 54 aa position, sited within the basic region of the RNA-binding domain. The multiple alignment has been done using MEGA X software. The dotted line separates the isolates according to this polymorphism.

As in a previous population study [22], the nucleotide sequence of the p23 gene was 99% homologous to those of SY and no-SY isolates belonging to the VT strain, with a single-nucleotide variation (G/A) at position 161 causing an amino acid change from serine to asparagine at position 54 and leading to a slight modification in the basic region of the RNA-binding domain (Figure 3). Multiple sequence alignment with CTV isolates representative of different strains showed that the VT, T36, T3, T318A isolates have a guanine at nucleotide position 161 and a serine at the amino acid level, similarly to VT SY isolates (SG29, N3 and Mac1a). On the contrary, T30, T385, RB have adenine and asparagine, respectively, similarly to VT no-SY isolates (M101, Mac39, M39D, M55, Q7 and N1) (Figure 3). Biological observations indicate that VT, T36, T3, and T318A can induce SY on sour orange and sweet orange grafted onto SO [46,47], while the T30, T385, and RB isolates are totally asymptomatic on SO. These observations confirm the role of the G/A-161 nt (Ser/Asn 54 aa) polymorphism in the shift from an SY to a no-SY phenotype in CTV isolates. 

### 3.3. Evaluation of SY and No-SY VT Variants via Real-Time RT-PCR and HRM Assay on p23 Gene

**Figure 4:**
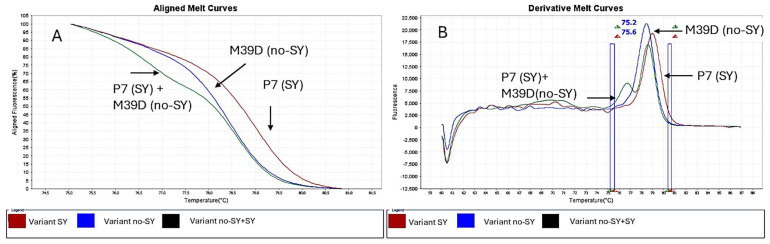
Aligned (**A**) and derivative melting curves (**B**) developed through a High-Resolution Melt Software v3.2 analysis of amplicons generated from RNAs extracted from P7 (SY) and/or M39D (no-SY) of partial p23 genes encompassing the G/A polymorphism at 161 nt. The red profile is associated with SY variants, the blue profile with no-SY variants, and the green profile with SY + no-SY variants. Green and red triangles indicate the minimum and maximum values of Pre-Melt and Post-Melt region range of temperature (blue rectangle).

A primer pair flanking the nucleotide of the p23 gene was evaluated by using RT-PCR high-resolution melting (HRM) to detect CTV-VT variants. RNA extracts from a total of six isolates representing SY and no-SY variants, based on SO seedlings’ reactions, were analyzed. Efficient amplification of the expected 78 bp fragment was achieved for each variant. The melting temperatures values generated by the HRM curve analysis were statistically different for SY (average 79.3 °C) and no-SY variants (average 78.4 °C), allowing their successful differentiation (Table 2, Figure 4). A third HRM profile was obtained by mixing RNAs extracted from plants inoculated with P7 and M39D isolates and showed two peaks: a main one at 78 °C Tm and a lower one at 77 °C (Figure 4).

To verify the assay′s robustness, a random inoculation of no-SY and SY variants was performed on eight rutaceous species, including grapefruit, sweet orange, volkameriana, and ornamental plants belonging to the genera *Microcitrus, Fortunella*, and *Atalantia* (Table 2). One-way ANOVA of the Tm values generated by HRM assays showed a statistically significant difference between the treatments (*p* < 0.001) (Table S3). Plants inoculated with no-SY variants revealed means of Tm values ranging between 78.28 °C and 78.64 °C, regardless of the host plant, the isolates and the year of infection, significantly different from the means of Tm values of SY variants ranging from 79.24 °C to 79.35 °C (Table 2). Two homogeneous groups were identified considering the variant (SY or no-SY) of the VT isolate used for inoculation. Within each group there are no statistically significant differences. The assay’s reproducibility and the stability of the isolates allow the use of HRM technology to discriminate between potential infections with no-SY and SY isolates in superinfected plants.

### 3.4. Real-Time RT-PCR and HRM Analysis of Cross-Protected Plants

**Figure 5:**
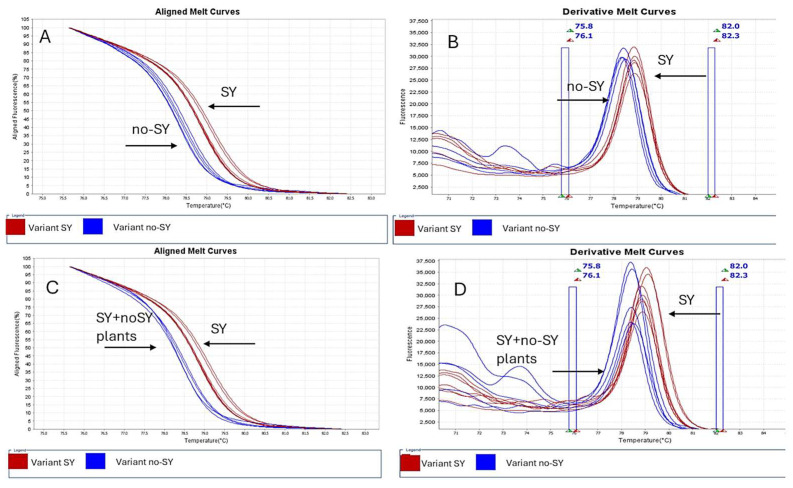
High-resolution melting curve profiles obtained for RNA extracted from plants inoculated with SY or no-SY isolates and from plants inoculated with SY and no-SY isolates, using High Resolution Melt Software v3.2 (Applied Biosystems TM, Waltham, MA, USA). Aligned melting curves (**A**,**C**) and derivative melting curves (**B**,**D**) were obtained using SYTO 9 for CTV variant detection. RNAs from plants inoculated with SY (in red) or no-SY isolates (in blue) are represented by (**A**,**B**). RNAs from plants inoculated with SY + no-SY isolates (in blue) are represented by (**C**,**D**). Green and red triangles indicate the minimum and maximum values of Pre-Melt and Post-Melt region range of temperature (blue rectangle).

Sour orange seedlings challenged with the P7 SY isolate 11 months after the first inoculation with no-SY M39D were tested by means of real-time RT-PCR and HRM analysis three years post-superinfection. The single- and double-infected plants did not show SY symptoms, and their performance was not dissimilar to that of the uninoculated control. One-way ANOVA of Tm values generated by HRM analyses of RNA extracts revealed statistically significant differences between the isolates used for inoculation (*p* < 0.001) (Table S4). Two homogeneous groups were identified considering the variant (SY or no-SY) of the VT isolate used for inoculation. In particular, according to results obtained for the Seq trial on SO, seedlings inoculated with solely P7 (SY) revealed an average melting temperature of 79.23 °C, significantly different from that of seedlings inoculated with M39D (no-SY) (Table 3, Figure 5). On the contrary, no significative differences were detected between seedlings pre-inoculated with M39D and challenged with P7 and those inoculated with only the no-SY variant (Table 3). Similar results with no statistically significant differences between the groups were obtained in plants pre-inoculated with Nan1 and M101 no-SY isolates and later challenged with P7 or P1R1 SY isolates, as revealed in the trials Nan (Nan1 + P7), H/AA (M39D + P7), CP (M101 + P1R1), and Mac (M39D + P1R1). The superinfected plants showed Tm values similar at a statistical level to that of plants inoculated with a no-SY isolate and different from plants inoculated with P7 (Table 3, Figure 5). No mixed profile associated with the co-presence of SY and no-SY variants was detected in the cross-protected plants. 

Based on these data, regardless of the host (SO or H/SO), it appears that the establishment of SY isolates was excluded in cross-protected plants, or their replication was repressed and maintained at an undetectable level four years post SY inoculation.

Furthermore, we extended the HRM assays to analyze the midveins and roots of SO seedlings inoculated 12 months prior with bark tissue taken from superinfected or not SO plants of the Seq and CP trials. One-way ANOVA of Tm values showed not significant differences between the plants analyzed (*p* = 0.35) (Table S5). As shown in Table 4, the average melting temperatures were between 78.32 °C and 78.72 °C with no statistically differences between the inoculation on the respective source plants. SO seedlings inoculated with tissues from plants single-inoculated with M39D or M101 no-SY isolates were statistically similar to that further challenged with P7 or P1R1 SY isolates. These data demonstrate that the SY isolate is not replicating in superinfected plants.

### 3.5. Variant Calling Analysis on p23 Gene of Cross-Protected Sour Orange Plants

In order to validate the results obtained via HRM, a variant calling analysis on the G/A polymorphism of the p23 gene was undertaken by means of deep sequencing of the amplicon using HTS. This method was applied to representative cross-protected plants to examine their sequence variations and associate them with the presence or absence of SY and no-SY CTV isolates. A total of six cross-protected plants (trials Seq and H/AA) were analyzed. The variant calling analysis involved about 1 million sequences, and the nucleotide frequency at position 161 of the p23 gene was determined. The allelic dominance of high-quality bases, considering both forward and reverse strands, showed a prevalence of ‘adenine’ (specific to no-SY isolates) with a frequency of about 99% across all the tested cross-inoculated plants (Table S6). On the contrary, a negligible presence of other nucleotides, including ‘guanine’ (specific to SY isolates), was identified. This result confirms that in cross-protected plants, only the no-SY isolate used for pre-inoculation is able to replicate, with no evidence of the P7 SY isolate used to challenge these plants.

## 4. Discussion

This study, which started more than ten years ago in a greenhouse [22] and has since been extended to containers maintained in open air, showed the stability of the cross-protection offered to sour orange and sweet oranges grafted onto sour orange trees by no-SY isolates against superinfection with homologous SY variants. Biological indexing of SO seedlings and Hamlin SwO grafted onto SO inoculated with no-SY isolates and superinfected with the P7 SY isolate confirmed the repression of yellowing [22]. After a long period post-inoculation—four years in open air—no symptoms appeared in the inoculated plants or in the indicator SO. Virus infection was detected by means of ELISAs and real-time PCR tests, indicating that the conditions were conducive to virus spread. The aim of the present study was to make some progress in elucidating the cross-protection mechanism, using different approaches. Thanks to a specifically designed HRM assay, it was possible to distinguish and specifically identify the two variants (SY and no-SY) in inoculated plants according to their different dissociation curves and melting temperatures. 

This test provided a valuable method to examine whether the primary infected variant could exclude the infection of a second variant of the same strain, and it demonstrated that an exclusion mechanism occurred in SO and Hamlin/SO cross-protected plants. Additionally, the absence of the SY variant used for the challenge inoculation was confirmed by the lack of a symptomatic reaction on SO seedlings artificially inoculated with tissue bark from superinfected plants.

These biological and molecular tests showed that the severe viral strain could not superinfect SO seedlings or Hamlin trees grafted onto SO preimmunized with homologous M39D and M101 VT no-SY variants of the same virus. Superinfection exclusion has also been observed in other strains (T36 and T68) and host plants (*Nicotiana benthamiana*) [48], suggesting that this cross-protection mechanism is typical of CTV. In *N. benthamiana*, it was hypothesized that an isolate of the T36 strain prevents subsequent infection by virulent RNA through localized RNAi or competition for host cell resources [49].

For CTV, the sequence homology between no-SY variants and superinfecting isolates is crucial, as the action relies on nucleic acids, which were not considered in earlier studies. The polymorphism at nucleotide position 161 of the p23 gene lies in a variable region linked to SY syndrome development by VT isolates on sour orange in greenhouse conditions [8], along with QD symptoms, and may serve as a useful marker for distinguishing between different SY variants. The position corresponds at the aminoacidic level to 54 aa, inducing a change from serine to asparagine in a region (50–54) sited within the basic region of the RNA-binding domain, close to the zinc finger domain [24]. Its role in the shift from an SY to a no-SY phenotype seems to be confirmed, although the mechanism remains unclear.

Folimonova et al. [50] found that superinfection exclusion (SIE) in CTV could be protein-mediated, mainly determined by the p33 protein and its interactions with others. The p33 protein acts as a CTV effector, reducing viral virulence [48]. While it may not be essential for cellular-level SIE, it mediates SIE at the organism level [50]. Therefore, SIE by CTV involves multiple complex mechanisms.

Although analysis of variants appears to be essential to the advancement of knowledge on cross-protection, the full mechanisms underlying virus exclusion remain unknown. Recent findings suggest that non-coding sgRNA and viral proteins play a key role in the virus infection cycle, systemic movement, and the host response [51]. Primary virus infection may prevent the entry of a secondary virus or induce host competition factors between viruses. 

The homology of protective and superinfective isolates can effectively be exploited to protect against citrus decline in regions where SO rootstock is more effective than alternatives. This may prevent yield losses and reduce agro-chemical use, as seen with SP isolates in countries using cross-protection [50]. Field trials will clarify the ecological and biological aspects of this protection’s durability and the potential risks to other potential host plants of the virus [52]. While cross-protection has been reported against phylogenetically different viruses, it is typically effective against closely related isolates of the same virus.

The attractive perspective of cross-protection to prevent SY damage lets to envisage a potential practical application in the nursery and suggests some additional aspects that must be considered to continue the study of the evolution of infections. Fu et al. [23] found that simultaneous inoculation of mild and severe CTV strains did not affect their establishment in sweet orange, and the severe strain dominated. This impact may be related to the different genotypes involved (T30 and SY568) or to simultaneous inoculation. No evidence exists for the long-term field application of strictly homogenous SY and no-SY isolates. HRM analysis has been proven to be able to detect the co-presence of no-SY and SY isolates in purposely mixed samples of infected tissue, with adenine and guanine being co-present at varying percentages. This could be useful in monitoring the possible presence of both variants in long-term trials in open fields. Moreover, the HRM assay can identify new protective homologous isolates for managing SY isolates, although they must be confirmed through bioassays and high-throughput sequencing (HTS). A long-term evaluation will clarify the evolution of the variants’ interplay in coinfected plants and the other factors involved. 

## 5. Conclusions

The use of mild CTV SP isolates to reduce the symptoms caused by severe isolates began in some countries in the last century with the aim of maintaining plant health and high-quality production. The selection of protective isolates was initially made based on their phenotypic reaction and, more recently, has been associated with genetic similarity. This approach has proven effective for certain variants of T36 and T68 strains when applied in areas infected by the same virus population. However, the mechanism of cross-protection remains unclear, and field investigations are still lacking. Additionally, field trials involving the interactions between isolates, often limited to non-sequenced CTV isolates, have primarily focused on the growth of trees. 

The HRM analysis developed in this study was shown to successfully discriminate the variants and monitor their replication in SO and sweet orange trees grafted onto SO. It could be effectively used to detect the potential replication of severe isolates before symptom development. This could encourage the implementation of field trials to define the long-term effects of mild-strain cross-protection, improve the understanding of the mechanism involved, and evaluate the impacts on consumers. The physiological effects of multiple infections could also be investigated.

## Figures and Tables

**Table 1:** The selected citrus tristeza virus VT isolates, SY and no-SY, used in this study, and the response on indicator host plants observed 12 months post-inoculation.

Isolate	GenBank	Symptoms on Indicator Host Plants			
		Sour Orange (‘SY’)	Mexican Lime (’VC’ and ‘SP’)	Duncan Grapefruit (‘SP’)	Sour Orange (‘SY’)
M101	MW689620	no-SY	+	+	+
M39D	OR387854	no-SY	+	+	+
M55	OP345183	no-SY	+	+	+
Q7	OM803129	no-SY	+	+	+
Nan1	P345182	no-SY	+	+	+
P7	KC748392	SY	+++	++	+++
Nan3	OP345181	SY	+++	++	+++
P1R1	/ *	SY	+++	++	+++

SY: seedling yellows; VC: vein clearing; SP: stem pitting; * not submitted; +: slight; ++: moderate; +++: severe.

**Table 2:** Melting points of no-SY and SY isolates from different host plants obtained by real-time RT-PCR HRM curve analysis of partial p23 gene.

VT Isolate		Host Plant	Year of Inoculation	HRM Tm °C
**ID**	**Variant**			
M39D	no-SY	*Citrus aurantium*	2020	78.37 a *
		*Microcitrus papuana*	2021	78.42 a
		*M. australasica*		78.28 a
		*Fortunella obovata*		78.36 a
		*Atalantia ceylanica*		78.54 a
M39D	no-SY	*C. paradisi*	2017	78.53 a
M101		*C. aurantium*	2020	78.64 a
M55				78.40 a
P7	SY	*C. aurantium* *C. sinensis*	2020	79.24 b
Nan3				79.35 b
P1R1		*C. volkameriana*	2018	79.30 b

* Means followed by different letters are significantly different based on a one-way ANOVA followed by a Student-Newman-Keuls post-hoc test at the 99% significance level.

**Table 3:** Melting points of plants inoculated with no-SY (M39D, Nan1, M101) and/or SY VT isolates (P7, P1R1) based on real-time RT-PCR HRM curve analysis of partial p23 gene.

Infected Source Plants			Melting Temperature (°C)				
Trial	Host	VT Isolate	Biological Replicas				Average
Seq	SO	M39D + P7	78.38	78.51	78.49	78.50	78.47 a *
		M39D	78.41	78.38	78.40	78.45	78.39 a
		P7	79.33	79.27	79.10	79.24	79.23 b
Nan	SO	Nan1 + P7	78.66	78.70	78.56	78.45	78.59 a
		Nan1	78.41	78.57	78.42	78.67	78.52 a
H/AA	H/SO	M39D + P7	78.43	78.35	78.32	78.55	78.41 a
		M39D	78.50	78.53	78.45	78.32	78.45 a
CP	SwO/Mac	M101 + P1R1	78.42	78.47	78.42	78.47	78.45 a
Mac	SwO/Mac	M39D + P1R1	78.53	78.31	78.50	78.24	78.40 a
		M39D	78.40	78.53	78.38	78.27	78.40 a

* Means followed by different letters are significantly different based on a one-way ANOVA fol-lowed by a Student-Newman-Keuls post-hoc test at the 99% significance level.

**Table 4:** Melting points of leaves or roots of sour orange seedlings, 12 months post-inoculation with bark tissues taken from cross-inoculated symptomless plants based on real-time RT-PCR HRM curve analysis of partial p23 gene.

Source Plants	VT Isolate		HRM Tm °C
Seq2	M39D (no-SY) + P7 (SY)	Midveins	78.72 a *
Seq8	M39D (no-SY) + P7 (SY)		78.64 a
Seq10	M39D (no-SY) + P7 (SY)		78.32 a
CP	M101 (no-SY) + P1R1 (SY)		78.54 a
Seq2R	M39D (no-SY) + P7 (SY)	Roots	78.34 a
Seq7R	M39D (no-SY)		78.40 a
Seq12R	M39D (no-SY)		78.42 a

* Means followed by the same letters are not significantly different based on a one-way ANOVA followed by a Student-Newman-Keuls post-hoc test at the 99% significance level.

## Data Availability

The raw sequences of the HTS datasets have been deposited to the National Center for Biotechnology Information (NCBI) Sequence Read Archive (SRA) under BioProject PRJNA1279388 and BioSample accessions SAMN49480673, SAMN49480674, SAMN49480675, SAMN49480676, SAMN49480677, SAMN49480678.

## Supplementary Materials

The following supporting information can be downloaded at https://www.mdpi.com/article/10.3390/pathogens14070701/s1, Table S1: Global list of host plants pre-inoculated with VT no-SY isolates, superinfected or not with SY, and not inoculated; Table S2: Accession numbers of full genome sequences of representative CTV strains used in the multiple alignment of p23 gene; Table S3: Analysis of Variance referred to Table 2 of the main text; Table S4: Analysis of Variance referred to Table 3 of the main text; Table S5: Analysis of Variance referred to Table 4 of the main text; Table S6: Allelic dominance of high-quality bases at nucleotide position of 161 of p23 gene determined by variant calling analysis on RNA extracts from cross-protected plants.
