# Supplementary material for: Progress in Our Understanding of the Cross-Protection Mechanism of CTV-VT No-SY Isolates Against Homologous SY Isolates
Source: Pathogens. 2025 Jul 16;14(7):701. doi: 10.3390/pathogens14070701 (PMC12297970; doi:10.3390/pathogens14070701)
Supplement: Supplementary file 1 [file pathogens-14-00701-s001.zip › pathogens-3625836-supplementary.pdf]

Table S1. Global list of host plants pre-inoculated with VT no-SY isolates, superinfected or not with SY, and not inoculated

Host plants	VT Isolates		Number of plants		
	No-SY	SY	no-SY+ SY	no-SY	Control
Hamlin/SO	M39D	P7	4	3	//
Hamlin/SO	M39D	P7	5	//	4
SO	M39D	P7	4	5	6
SO	Q7	P7	6	//	3
SO	Nan1	P7	5	4	3
SO	M101	P1R1	3	3	3
Total	4	2	27	15	19

Table S2. Accession numbers of full genome sequences of representative CTV strains used in the multiple alignment of p23 gene

Genbank accession number	Isolate	Strain	Country	Host	Release date
EU937519	FS2-2	VT	Florida, USA	*C. sinensis* cv Hamlin	2016
DQ151548	T318A	VT	Spain	*Citrus sinensis*	2016
AF260651	T30	T30	Florida, USA	*C. sinensis* cv Valencia	2016
Y18420	T385	T30	Spain	*C. sinensis*	2016
KC525952	T3	T3	USA	*C. macrophylla*	2013
EU937521	FS2-2	T36	Florida, USA	*C. sinensis* cv Hamlin	2016
MZ870354	RB	RB	Ecuador	Lemon Meyer	2022
KCT748392	SG29	VT	Italy	*C. sinensis* cv Sanguinello	2015
MW689620	M101	VT	Italy	*C. macrophylla*	2021
KJ790175	M39	VT	Italy	*C. macrophylla*	2016
OR387854	M39D	VT	Italy	*C. paradisi* cv Duncan	2023
OP345183	M55	VT	Italy	*C. macrophylla*	2023
OM803129	Q7	VT	Italy	*C. sinensis* cv Tarocco	2023
OP345182	N1	VT	Italy	*C. sinensis* cv Tarocco	2023
OP345181	N3	VT	Italy	*C. sinensis* cv Tarocco	2023
Not submitted	Mac1a	VT	Italy	*C. macrophylla*	-

Table S3. Analysis of Variance referred to Table 2 of the main text

Source	Sum of Squares	Df	Mean Square	F-Ratio	P-Value
Between groups	5,19456	11	0,472233	32,45	$1.02x10^{-11}$
Within groups	0,349267	24	0,0145528		
Total (Corr.)	5,54383	35			

Table S4. Analysis of Variance referred to Table 3 of the main text

Source	Sum of Squares	Df	Mean Square	F-Ratio	P-Value
Between groups	2.33167	9	0.259075	27,64	$3.8x10^{-12}$
Within groups	0.281225	30	0.00937417		
Total (Corr.)	2.6129	39			

Table S5. Analysis of Variance referred to Table 4 of the main text

Source	Sum of Squares	Df	Mean Square	F-Ratio	P-Value
Between groups	0.2854	6	0.0475667	1.36	0.3470
Within groups	0.2456	7	0.0350857		
Total (Corr.)	0.531	13			

Table S6. Allelic dominance of high-quality bases at nucleotide position of 161 of p23 gene determined by variant calling analysis on RNA extracts from cross-protected plants.

				A	T	C	G
Trial	Sample	Host	Isolates	Frequency (%) / Coverage (x)			
Seq	seq2	SO	M39D + P7	99.83/95,711	0.03/29	0.01/10	0.13/125
	seq8	SO	M39D + P7	99.79/95,587	0.04/38	0.01/10	0.16/153
	seq10	SO	M39D + P7	99.91/18,401	0/0	0.01/2	0.09/17
H/AA	H/AA 6	SwO/SO	M39D + P7	99.81/92,902	0.01/9	0/0	0.18/168
	H/AA 11	SwO/SO	M39D + P7	90.06/83,915	0.01/9	0.01/9	9.93/9,252
	H/AA 13	SwO/SO	M39D + P7	99.45/93,124	0/0	0.01/9	0.54/506
